# Elastic Liposomes Containing Calcium/Magnesium Ferrite Nanoparticles Coupled with Gold Nanorods for Application in Photothermal Therapy

**DOI:** 10.3390/nano14080679

**Published:** 2024-04-15

**Authors:** Ana Rita F. Pacheco, Ana Margarida Barros, Carlos O. Amorim, Vítor S. Amaral, Paulo J. G. Coutinho, Ana Rita O. Rodrigues, Elisabete M. S. Castanheira

**Affiliations:** 1 Physics Centre of Minho and Porto Universities (CF-UM-UP), University of Minho, Campus de Gualtar, 4710-057 Braga, Portugal; anaritapacheco99@gmail.com (A.R.F.P.); pg42479@alunos.uminho.pt (A.M.B.);; 2 Associate Laboratory LaPMET, Campus de Gualtar, 4710-057 Braga, Portugal; 3 Physics Department and i3N, University of Aveiro, Campus de Santiago, 3810-193 Aveiro, Portugal; 4 Physics Department and CICECO, University of Aveiro, Campus de Santiago, 3810-193 Aveiro, Portugal

**Keywords:** calcium/magnesium ferrite, gold nanorods, elastic liposomes, photothermia

## Abstract

This work reports on the design, development, and characterization of novel magneto-plasmonic elastic liposomes (MPELs) of DPPC:SP80 (85:15) containing Mg_0.75_Ca_0.25_Fe_2_O_4_ nanoparticles coupled with gold nanorods, for topical application of photothermal therapy (PTT). Both magnetic and plasmonic components were characterized regarding their structural, morphological, magnetic and photothermal properties. The magnetic nanoparticles display a cubic shape and a size (major axis) of 37 ± 3 nm, while the longitudinal and transverse sizes of the nanorods are 46 ± 7 nm and 12 ± 1.6 nm, respectively. A new methodology was employed to couple the magnetic and plasmonic nanostructures, using cysteine as bridge. The potential for photothermia was evaluated for the magnetic nanoparticles, gold nanorods and the coupled magnetic/plasmonic nanoparticles, which demonstrated a maximum temperature variation of 28.9 °C, 33.6 °C and 37.2 °C, respectively, during a 30 min NIR-laser irradiation of 1 mg/mL dispersions. Using fluorescence anisotropy studies, a phase transition temperature (T_m_) of 35 °C was estimated for MPELs, which ensures an enhanced fluidity crucial for effective crossing of the skin layers. The photothermal potential of this novel nanostructure corresponds to a specific absorption rate (SAR) of 616.9 W/g and a maximum temperature increase of 33.5 °C. These findings point to the development of thermoelastic nanocarriers with suitable features to act as photothermal hyperthermia agents.

## 1. Introduction

In modern medicine, liposomes have emerged as ideal and effective nanocarriers. These well-studied biocompatible vesicles ensure the protection, stabilization and transport of different substances, both hydrophilic and hydrophobic, without changing their mechanism of action [1,2]. Considering the treatment of skin diseases, which represent one of the most frequent types of disorders all over the world [3], liposomes must be designed to ensure high penetration into the different layers of the skin, especially into its barrier, the stratum corneum (SC). For that purpose, elastic liposomes (ELs) are an excellent approach for topical applications due to their ultra-deformable properties [4,5,6]. Generally, ELs consist of an aqueous core surrounded by a double bilayer of phospholipids and single chain surfactants with a high radius of curvature, also known as edge activators (EAs) [1,7]. These are responsible for reducing the phase transition temperature and conferring an elastic nature to liposomes by modulating their interfacial tension and forcing the reorganization of the main phospholipid, increasing their flexibility. Thus, when a transdermal water gradient occurs, the high deformability of ELs allows them to pass spontaneously across the SC, without modifying their initial structure [7]. In fact, vesicles with this behavior manage to penetrate channels ten times smaller than their diameter, allowing them to reach the SC by the intercellular route [8]. Typical EAs include oleic acid, polysorbate (Tween), sorbitan (Span) and sodium cholate [9]. For the main phospholipid composition, lipids containing phosphatidylcholine with a T_m_ localized above the physiological temperature are usually included in the formulations. In this context, dipalmitoyl-phosphatidyl-choline (DPPC) shows great potential (T_m_ ≃ 41 °C) [10]. Below this value, liposomes are in a solid gel phase. However, with the temperature increase, they will attain a fluid liquid crystal state, and an enhanced permeability will be promoted, releasing their content [9]. Vesicles exhibiting this behavior are known as thermosensitive liposomes. Their conjugation with EAs leads to a reduction in the liposome’s transition temperature. Considering topical applications, it is important that a system’s T_m_ is adjusted and localized between 25 °C (room temperature) and 35 °C (average skin temperature [11]) for an improved permeation potential and stability, by optimizing the ingredients of elastic liposomes, as well as the lipid/EA ratio.

After ensuring topical administration through ELs, other strategies are required for skin disease therapy. On the one hand, the incorporation of magnetic nanoparticles (MNPs) into the nanoplatform is a beneficial solution, because with proper composition they can behave as multi-modal therapeutic agents (from diagnosis and magnetic targeting to drug delivery and hyperthermia mediators) [12]. To avoid toxicity issues, the biocompatibility of the MNPs must be ensured. Considering this, ferrites containing alkaline earth metals, such as Mg^2+^ and Ca^2+^ ions, are preferred for inclusion in the final formulation, as a result of their proved cell viability [13]. Promising previous results using magnesium doped ferrite nanoparticles have been reported, in particular for Ca_0.25_Mg_0.75_Fe_2_O_4_ MNPs. These have shown a superparamagnetic behavior and high thermal energy dissipation, when irradiated with near-infrared (NIR) light or by exposure to an alternating magnetic field [13,14,15,16]. To obtain enhanced properties, and especially a more efficient hyperthermia capability, shape anisotropic MNPs have proven to be a good option [17,18,19,20].

On the other hand, gold nanorods (AuNRs) can provide an even more pronounced photothermal conversion efficiency [21,22,23,24]. When irradiated by a particular frequency of light equal to the inherent frequency of the conductive electrons of AuNRs, these start to collectively oscillate at the nanorods surface, reaching their maximal amplitude of oscillation. This results in the free electrons’ resonance, a phenomenon called localized surface plasmon resonance (LSPR) [24]. Since they show two LSPR bands (transverse and longitudinal), rod-shaped gold nanoparticles exhibit excellent light absorption in the NIR spectral region (650–1100 nm), which translates into suitable photothermal agents with an efficient body penetration and notable temperature rise at target tissues [25].

The incorporation of magnetic/plasmonic nanoparticles (MPNPs) in the aqueous interior of lipid vesicles, forming magneto-plasmonic elastic liposomes, is a promising approach with potential to treat topical disease conditions through hyperthermia. The combination of both magnetic and plasmonic components in a single system can maximize the cell damage by an abnormally localized heat under a NIR laser (plasmonic hyperthermia) [26,27]. Several magnetic/plasmonic nanostructures have been reported, most of them using ferrites as the magnetic component (e.g., iron oxide, manganese ferrite) and gold or carbon as the plasmonic element [28,29,30].

Herein, we propose a novel nanosystem capable of action as magnetic and plasmonic hyperthermia agent at low concentration for skin disease therapy. For this purpose, Mg_0.75_Ca_0.25_Fe_2_O_4_ nanocubes were coupled with Au nanorods and surrounded by a lipid bilayer of DPPC:SP80 (85:15) with elastic properties for improved penetration in the SC. The response of MPELs and their components to external stimuli was thoroughly examined, in order to determine their heating capability, along with investigating the impact of the interaction between MNPs and AuNRs. To the best of our knowledge, the coupling between Ca/Mg mixed ferrite nanocubes and Au nanorods has not previously been reported in the literature, as well as their encapsulation in elastic liposomes. Promising results were obtained for MPELs’ future application in skin disease treatment by photothermal hyperthermia.

## 2. Materials and Methods

The chemicals iron(III) chloride hexahydrate, magnesium acetate tetrahydrate, calcium acetate hydrate, cis-9-octadecenoic acid (OA), tetrahydrofuran (THF), 1-octadecene, chloroform, hydrogen peroxide, sodium borohydride, dimethyl sulfoxide 99.9% (DMSO), gold(III) chloride solution, cetyltrimethylammonium bromide (CTAB), silver nitrate, hydrazine monohydrate, DL-cysteine 97%, imidazole hydrochloride, potassium phosphate monobasic, hydroquinone, sodium sulphate anhydrous, 1,1′-carbonyldiimidazole (CDI), sorbitan monostearate (SP60), sorbitan tristearate (SP65), sorbitan monooleate (SP80), sorbitan trioleate (SP85) and poly-oxyethylene-sorbitan monooleate (TW80) were purchased from Sigma-Aldrich (St. Louis, MO, USA). 1,2-Dipalmitoyl*-sn*-glycero-3-phosphocholine (DPPC) was purchased from Avanti Polar Lipids (Birmingham, AL, USA). Spectroscopic grade solvents and ultrapure water Milli-Q grade (MilliporeSigma, St. Louis, MO, USA) were used in all preparations.

### 2.1. Synthesis of Mg_0.75_Ca_0.25_Fe_2_O_4_ Nanocubes

Calcium/magnesium mixed ferrite nanocubes were prepared by an adapted protocol from Pacheco et al. [31], which involves the use of the surfactants octadecene and oleic acid as solvent and reducing agent, respectively. OA plays an important role in the surface mediated phase transfer of the pre-generated spherical nanoparticles to new crystal structures during the growth and nucleation stages [32].

To prepare cubic-shaped magnetic nanoparticles of magnesium ferrite with 25% replacement by calcium ions, a solution containing 3.1 mM oleic acid, 2 mM iron(III) chloride hexahydrate, 0.75 mM magnesium acetate tetrahydrate and 0.25 mM calcium acetate hydrate in 15 mL of octadecene was heated to 120 °C, under continuous magnetic stirring. After 1 h at these conditions, a condenser was attached to the system and the temperature was increased at a rate of 1 °C per min until reaching 200 °C. After 1 h, the mixture was heated at a rate of 5 °C per min until 290 °C and then left at this temperature for 60 min. The precipitated MNPs were washed by several cycles of magnetic decantation with THF and a solution of water/ethanol (1:1). In order to remove surface surfactant residues, a chemical calcination step was performed using an adapted protocol previously described [33]. Briefly, the total of nanoparticles was treated with a 35% hydrogen peroxide (H_2_O_2_) solution, in a proportion of 1 g of MNPs per 40 mL of solvent, for 48 h at room temperature, under continuous magnetic stirring. Considering this exothermic reaction, an ice bath was used. Finally, the mixed ferrite nanocubes were washed with ethanol and water by magnetic decantation and then dried.

### 2.2. Synthesis of Gold Nanorods

Gold nanorods (AuNRs) were synthesized using a chemical reduction protocol adapted from [34]. Briefly, an aqueous solution of 9141 µL containing 364.45 mg of CTAB was prepared and heated up to a temperature of 30-35 °C in a water bath, with magnetic stirring. Subsequently, 400 µL of gold(III) chloride solution (0.01 M), 75 µL of silver nitrate (0.15 mM), 364 µL of hydroquinone (0.14 M) and 20 µL of sodium borohydride (0.005 M) were added. All additions were performed within 5 min. intervals. During each interval, the next solution to be added was prepared. After 30 s following the last addition, magnetic stirring was removed, and the solution was maintained at 35 °C for 4 h.

The obtained nanoparticles were washed by centrifugation (10,000 rpm for 30 min), using a 2 mM CTAB solution. In order to promote the deposition of the gold nanorods, 50 µL of a monopotassium phosphate solution (1 M) were added. Finally, the purified nanoparticles were dispersed in a 2 mM CTAB solution for future use.

### 2.3. Coupling between MNPs and AuNRs

**Figure 1:**
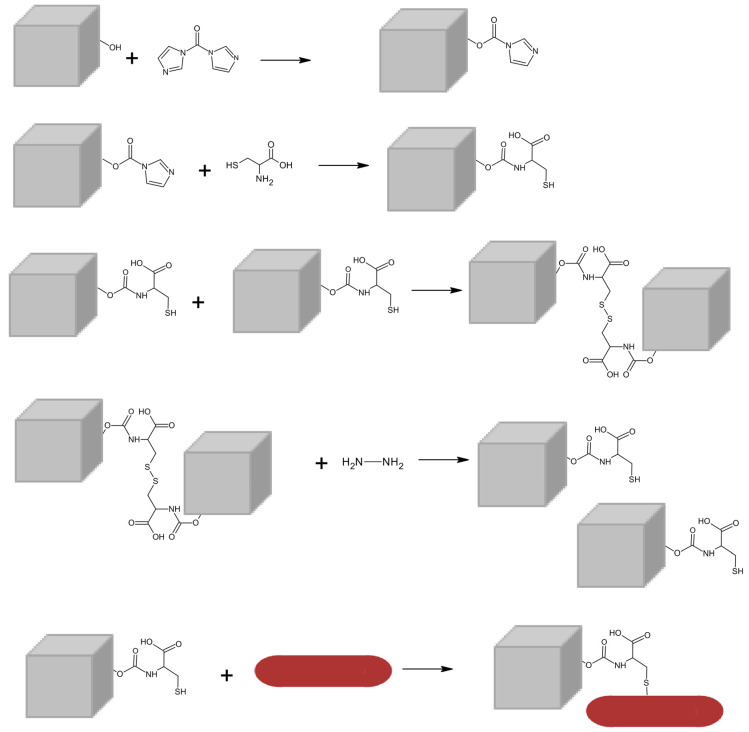
Schematic representation of the coupling between Mg_0.75_Ca_0.25_Fe_2_O_4_ nanocubes and gold nanorods, originating magneto-plasmonic nanoparticles as the final structure.

Magneto-plasmonic nanoparticles were obtained by coupling Ca/Mg mixed ferrite nanocubes and gold nanorods. Cysteine was employed as a “bridging” molecule between the two types of nanoparticles (Figure 1). The –OH groups at the surface of ferrite nanocubes were first activated with carbonyl-diimidazole (CDI). Through the amine group (–NH_2_) in cysteine, a carbamate linkage was formed. The pending thiol groups (–SH) of cysteine can strongly chemisorb to the gold surface, resulting in the coupling of the two types of nanoparticles. Hydrazine is additionally used to prevent aggregation of the cysteine functionalized ferrite nanocubes through formation of S–S bonds.

First, a 0.2 mg/mL solution of magnetic nanoparticles in dry dimethyl-sulfoxide (DMSO) was prepared. Subsequently, 3.14 mg of CDI was added, and the mixture was heated up to 60 °C, under magnetic stirring, and kept overnight at these conditions. The nanoparticles were subjected to three washing cycles with a mixture of ethanol/water (1:1) by magnetic decantation, to remove any remaining CDI molecules. Upon redispersion in dried DMSO, 2.5 mg of cysteine were added and 2.17 mg of imidazole-HCl was used as a reaction catalyst. The mixture was allowed to react for 1 h at the same temperature (60 °C) under magnetic stirring, and the washing procedure was repeated. The final step was the addition of 5 mL of a pre-prepared solution of gold nanorods along with 20 µL of hydrazine solution (8.24 × 10^−4^ M). The reaction was maintained for 1 h in the same conditions. Finally, the magneto-plasmonic NPs were washed by magnetic decantation to remove all gold nanorods that did not attach to the magnetic nanoparticles. The supernatant and the sediment (coupled nanoparticles) were redispersed in 5 mL of 0.2 mM CTAB.

### 2.4. Preparation of Elastic Liposomes and Aqueous Elastic Magnetoliposomes

Several lipid formulations using DPPC as the main phospholipid and with different surfactants, namely Span-60 (SP60), Span-65 (SP65), Span-80 (SP80), Span-85 (SP85) and Tween-80 (TW80), were prepared. Different molar ratios of 70:30, 80:20 and 85:15 were investigated. The conventional ethanolic injection method was followed in the preparation of elastic liposomes, as described by Ribeiro et al. [35]. Briefly, an ethanolic lipid solution of 1 mM was injected, drop by drop and under continuous stirring, to a pre-heated ultrapure water (55 °C), inducing the vesicles’ formation.

Aqueous elastic magneto-liposomes were prepared following a similar procedure, using the lipids DPPC and SP80 (85:15) in a final concentration of 1 mM. For this purpose, the lipid content was injected, under vortex, to 3 mL of an aqueous dispersion of magneto-plasmonic nanoparticles (1 × 10^−4^ M) above the main phospholipid phase transition temperature (55 °C). The non-encapsulated nanoparticles were removed by several cycles of magnetic decantation [36].

### 2.5. Structural and Magnetic Characterization

The composition and crystallographic structure of the mixed ferrite nanocubes were investigated by X-ray diffraction (XRD) using a PAN’alytical X’Pert PRO diffractometer (Malvern Panalytical Ltd., Malvern, UK), at the Electron Microscopy Unit of the University of Trás-os-Montes and Alto Douro (UTAD), Vila Real, Portugal. The sample was analysed operating with CuK_α_ radiation (λ = 0.154060 nm), in a Bragg–Brentano configuration. The XRD diffractogram was acquired and interpreted using Profex Software (version 4.3.6) (RMS Foundation, Bettlach, Switzerland) [37], with a BGMN Rietveld model [38].

Transmission electron microscopy (TEM) images of isolated and coupled magnetic and gold nanoparticles were recorded at the Centro de Apoio Científico-Tecnolóxico à Investigación (CACTI), University of Vigo, Spain, using a JEOL JEM-1010 high-contrast microscope, operating at 100 kV. The aqueous elastic magneto-plasmonic liposomes were also observed by TEM. Copper grids with carbon and Formvar were used for sample preparation and the NPs’ dispersion was ultrasonicated before deposition. The images were processed using ImageJ software (version 1.53t, National Institutes of Health (NIH), Bethesda, MD, USA). Average sizes were estimated using the length and height of each particle and the corresponding histogram was fitted to a Gaussian distribution.

Dynamic light scattering (DLS) and electrophoretic light scattering (ELS) techniques were employed to determine the average hydrodynamic size and colloidal stability in the Litesizer^TM^ 500 DLS equipment from Anton-Paar (Anton-Paar GmbH, Graz, Austria) containing a laser diode of λ = 658 nm. Three independent measurements were performed for each sample, at room temperature, and the experimental data were processed using Kalliope software (Anton-Paar GmbH, Graz, Austria).

Magnetic response of Mg_0.75_Ca_0.25_Fe_2_O_4_ nanocubes and magneto-plasmonic nanoparticles was assessed at University of Aveiro (Portugal) using a MPMS3 Superconducting Quantum Interference Device (SQUID) magnetometer Quantum Design MPMS5XL (Quantum Design Inc., San Diego, CA, USA). The measurements were conducted at room temperature with applied magnetic fields in the convenient range for each sample. A specific magnetic field correction for the trapped flux in the superconducting coil was performed, resulting in an accuracy of residual less than 2 Oe. Corrections were applied to the results for geometric effects and additional measurements were made to rectify the remaining field of the superconducting coil.

### 2.6. Measurement of Hyperthermia Capability

The ability for photothermal therapy was evaluated using a home-made experimental irradiation setup at the Physics Centre of University of Minho (Braga, Portugal). This setup is equipped with a sample holder, a continuous laser light source with λ = 808 nm and 1 W/cm^2^ of power density, and a T-type thermocouple connected to a digital multimeter (Agilent U1242A) for temperature measurement. The thermal energy dissipation of gold nanorods, magnetic NPs and magneto-plasmonic NPs dispersions (1 mg/mL) under NIR radiation was recorded for a period of time of 30 min. After this time, the laser was turned off and the cooling curves were recorded for another 30 min. The same procedure was employed for MPEL suspensions. For each sample, three independent measurements were performed.

The nanoparticles’ ability to release heat when irradiated with NIR light was evaluated by the specific absorption rate (SAR). This is defined as the total energy absorbed per unit of mass (W/g), and is given by Equation (1),

(1) $$SAR=C\frac{\Delta T}{\Delta t}\times \frac{m_{s}}{m_{m}}$$

where C is the specific heat capacity of the solution (4.186 J g^−1^ K^−1^ for water), ΔT/Δt expresses the initial slope of the curve and m_s_ and m_m_ refer to the mass of the solution and of the magnetic/plasmonic content (g), respectively [39]. The SAR calculation was performed employing the initial linear slope method.

### 2.7. UV-Vis–NIR and Fluorescence Measurements

UV-Vis–NIR absorption spectra were obtained on a double-beam Shimadzu UV-3600 Plus UV-Vis–NIR spectrophotometer (Shimadzu Corporation, Kyoto, Japan). Fluorescence steady-state anisotropy studies on the ELs and MPELs were performed using a Fluorolog 3 spectrofluorometer (HORIBA Jobin Yvon IBH Ltd., Glasgow, UK), possessing double monochromators (excitation and emission) and Glan–Thompson polarizers. To acquire these spectra, the probe 1,6-diphenyl-1,3,5-hexatriene (DPH) was included in all the formulations, at a final concentration of 1 × 10^−6^ M. The prepared samples were excited at λ_exc_ = 350 nm, and the temperature varied between 25 °C and 55 °C, using a temperature-controlled cuvette holder. To assess the apparent microviscosity (η), the steady-state fluorescence anisotropy (r) was calculated according to Equation (2):

(2) $$r=\frac{I_{VV}-{GI}_{VH}}{I_{VV}+{2GI}_{VH}}$$

where I_VV_ and I_VH_ correspond to the fluorescence intensities obtained with vertical (V) and horizontal (H) polarization, for vertically polarized excitation light, respectively, and G is the instrument correction factor. The latter is given by Equation (3),

(3) $$G=\frac{I_{HV}}{I_{HH}}$$

where I_HV_ and I_HH_ are the fluorescence intensities obtained with V and H polarization, for horizontally polarized excitation light, respectively. Considering a fluorescence lifetime for DPH of 11.4 ns in lipid membranes at room temperature [40,41], it is then possible to calculate η (P) from r values, following Equation (4) [40,41]. Three independent measurements were made, and the respective standard deviation (SD) was determined.

(4) $$\eta =\frac{2.4\;r}{0.362-r}$$

## 3. Results and Discussion

### 3.1. Magnetoplasmonic Nanoparticles’ Characterization

Magneto-plasmonic nanoparticles, i.e., Mg_0.75_Ca_0.25_Fe_2_O_4_ nanocubes coupled with gold nanorods, were prepared. Magnesium/calcium ferrites have the advantage of enhanced biocompatibility and of forming cubic-shaped nanoparticles that possess better magnetic properties than the corresponding spherical nanoparticles [15], as already reported for shape-anisotropic nanoparticles of the same composition [17]. A comprehensive characterization of each particle was conducted, as well as the final nanostructure, in order to study individual properties and analyze the effectiveness of the coupling.

**Figure 2:**
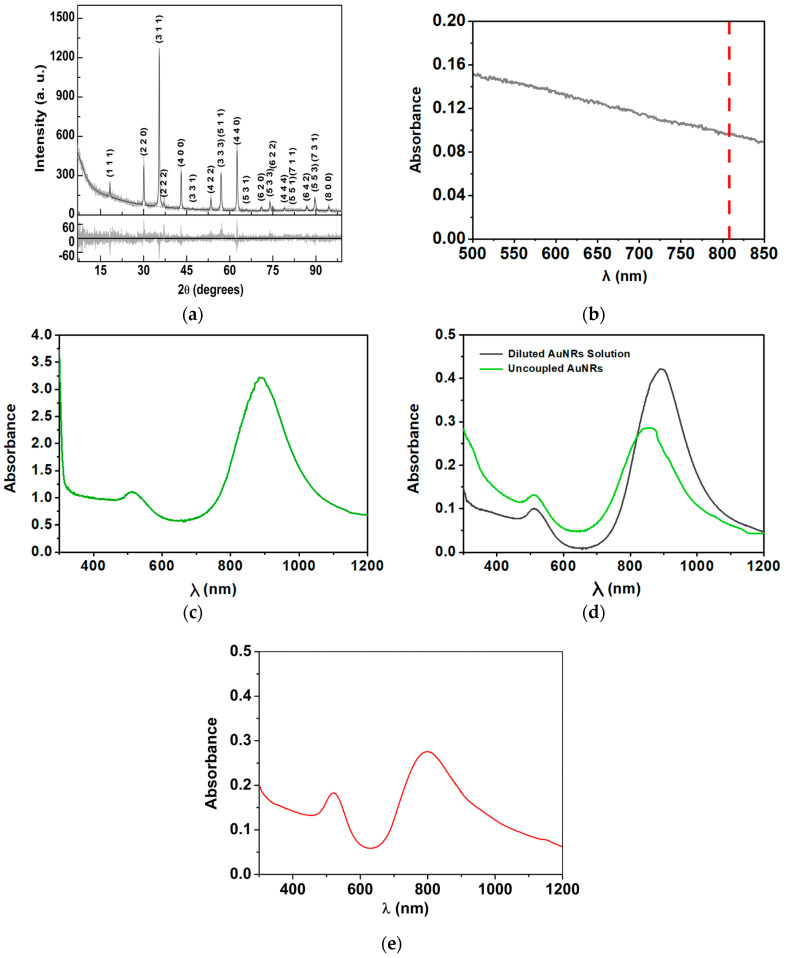
(**a**) X-ray diffractogram of Mg_0.75_Ca_0.25_Fe_2_O_4_ nanoparticles with the respective Rietveld analysis and Miller indices. The residuals of the fitting are displayed in the bottom panel. (**b**) UV-Vis–NIR absorption spectra of Mg_0.75_Ca_0.25_Fe_2_O_4_ nanoparticles dispersed in water (red line highlights absorption at 808 nm). (**c**) UV-Vis–NIR absorption spectra of gold nanorods dispersed in CTAB. (**d**) UV-Vis–NIR absorption spectra of diluted AuNRs solution and uncoupled AuNRs. (**e**) UV-Vis–NIR absorption spectrum of the coupled magneto-plasmonic nanoparticles.

The XRD pattern of the magnesium ferrite MNPs with 25% replacement by calcium ions was processed by importing the CIF file with number 1011245 (space group 227), from the crystallographic open database (COD). Since this file is valid for MgFe_2_O_4_, adjustments to the unit cell composition were implemented to correspond with the actual structure of the NPs. More precisely, 25% of the crystallographic positions occupied by magnesium ions were considered to be the calcium ions’ contribution. A variable inversion degree was assumed, considering that the distribution of Ca^2+^ and Mg^2+^ cations between tetrahedral and octahedral sites occurs so that the mixed ferrite stoichiometry is valid in both type of position. This led to a fit with χ^2^ = 1.341, R_P_ = 12.31 with an inversion degree of 0.5. The obtained diffractogram (Figure 2a) provides evidence for the synthesis of Mg_0.75_Ca_0.25_Fe_2_O_4_ nanoparticles, revealing an average size of 41.3 nm, estimated by analyzing all the diffraction peaks, as implemented in BGMN model [38]. These peaks appeared at 2θ = 18.4° (1 1 1), 30.2° (2 2 0), 35.6° (3 1 1), 37.2° (2 2 2), 43.3° (4 0 0), 47.4° (3 3 1), 53.7° (4 2 2), 57.2° (5 1 1), 57.2° (3 3 3), 62.9° (4 4 0), 66.1° (5 3 1), 71.3° (6 2 0), 74.4° (5 3 3), 75.4° (6 2 2), 79.4° (4 4 4), 82.4° (7 1 1), 82.4° (5 5 1), 87.2° (6 4 2), 90.2° (7 3 1), 90.2° (5 5 3), 95.1° (8 0 0), 98.0° (7 3 3), corresponding to a lattice constant *a* = 8.392 Å.

Figure 2b,c present the absorption spectra of Mg_0.75_Ca_0.25_Fe_2_O_4_ nanoparticles and gold nanorods, respectively. The magnetic nanocube spectrum (Figure 2b) reveals a broad absorption, showing that the MNPs can absorb light at 808 nm (as highlighted by the red line). Absorption of MgFe_2_O_4_ in the NIR region was reported as arising from intra-atomic transitions among the 3d energy levels split by the crystal field [42]. This type of transitions is known to be forbidden for Fe^3+^ (d^5^ high spin), but gains intensity through spin coupling interactions between Fe^3+^ atoms in close proximity [43]. At NIR wavelengths, the human tissues show minimal light absorption, which is a key feature for the main objective of this work. Several studies have documented a considerable photothermal potential exhibited by magnetic NPs under NIR laser irradiation [44,45,46], sparking significant interest and promise for the application of Ca/Mg ferrites in PTT. In fact, this aptitude has already been attested on different ferrite nanoparticles, being especially intensified in those based on alkaline earth metals [16,47,48].

Regarding the gold nanorods, the UV–Vis–NIR spectrum (Figure 2c) allows for conclusiio of their synthesis and the obtaining of information about their size and shape. Two absorption bands, the first around 500–550 nm and the second with a maximum at 880 nm, corresponding to the transverse plasmon and the longitudinal surface plasmon, respectively, are observed. Considering the position of the plasmonic bands, nanoparticles with a diameter of around 9 nm and length of 31 nm, are expected [34]. From these values (Figure 2c), gold nanorods with a diameter/length ratio of 3.5 are anticipated.

The coupling process of gold nanorods with Mg_0.75_Ca_0.25_Fe_2_O_4_ nanoparticles, allowing the obtaining of the magnetic and plasmonic components in a single nanosystem, was monitored by UV–Vis–NIR absorption spectroscopy. To these measurements, the gold solution was diluted to the same concentration that would be obtained in the supernatant solution, if no coupling has occurred. This solution was labeled as “diluted AuNR solution”. The absorption spectra of this solution and of the supernatant containing the uncoupled AuNRs are presented in Figure 2d. From the obtained spectra, it was possible to conclude that the intensity of the bands of gold nanoparticles that did not couple with the magnetic nanoparticles is lower than that which would have been obtained if no coupling has happened. This is an indication that the coupling has effectively occurred. The spectrum of coupled nanoparticles is displayed in Figure 2e, evidencing the coupling by the very different relation between the transverse and longitudinal plasmon bands.

The formation of magneto-plasmonic nanoparticles was assessed through the difference in maximum longitudinal plasmon absorbance of the initial gold nanorods used for coupling, designated as “diluted AuNR solution”, and the maximum absorbance of the longitudinal plasmon of gold nanoparticles that did not bound to the magnetic nanoparticles, designated as “uncoupled AuNRs”. Thus, the mass of bound gold was determined using Equation (5), corresponding to a coupling efficiency of 32%.

(5) $$\frac{Abs\left(diluted\;AuNRs\;solution\right)-Abs\;(uncoupled\;AuNRs)}{Abs\;(diluted\;AuNRs\;solution)}\times diluted\;AuNRs\;solution\;mass$$

**Figure 3:**
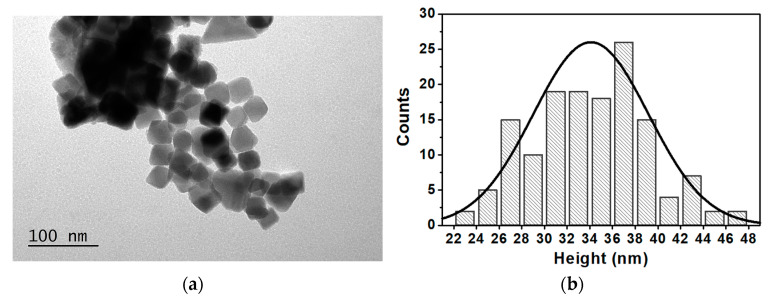

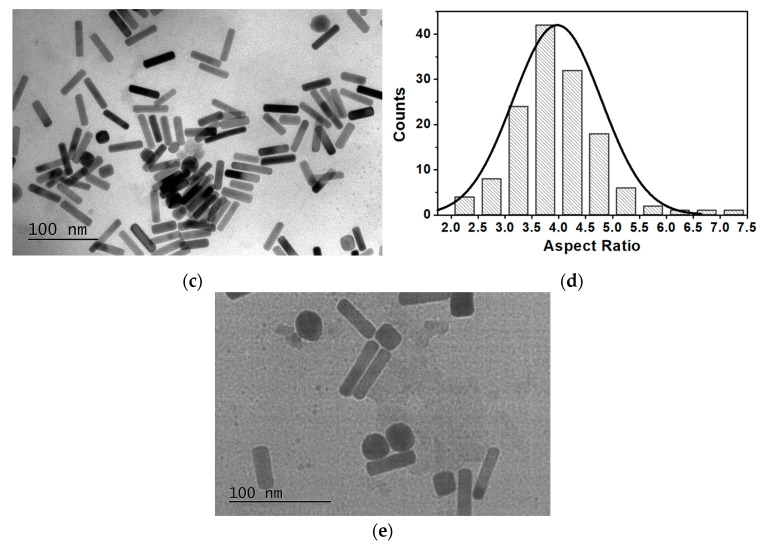
(**a**) TEM image of Mg_0.75_Ca_0.25_Fe_2_O_4_ nanocubes and (**b**) respective size histogram fitted to a Gaussian distribution. (**c**) TEM image of gold nanorods and (**d**) respective aspect ratio histogram fitted to a Gaussian distribution. (**e**) TEM image of magnetic nanoparticles coupled with plasmonic nanorods. Scale bar: 100 nm.

For accurate evaluation of the morphology and size distribution of magnetic and plasmonic NPs and of the coupling between them, a TEM characterization was performed. The obtained images are presented in Figure 3. The TEM images are in accordance with the expected, revealing the homogeneity of the prepared NPs and corroborating the successful coupling between the magnetic and plasmonic components, as predicted by the spectroscopic results. One the one hand, the mixed ferrite NPs generally exhibit a cubic shape (Figure 3a), with an average length of major and minor axes of 37 ± 3 nm and 31 ± 5 nm, respectively (Figure 3b), resulting in an aspect ratio ranging from 1.1 to 1.2. This value slightly above 1.0 reveals the presence of magnetic structures with a slight elongation, although some more spherical nanoparticles are also observed [17]. The results are in reasonable accordance with the average size estimated by XRD. One the other hand, the images obtained for plasmonic nanoparticles confirmed the formation of gold NPs in the shape of nanorods. Additionally, it is observed that these are uniform in shape and size. From Figure 3c, the distributions of longitudinal and transverse sizes of the nanorods were estimated as 46 ± 7 nm and 12 ± 1.6 nm, respectively. An aspect ratio of around 3.9 was calculated (Figure 3d), in general accordance with that obtained from UV–Vis–NIR absorption. This characterization was crucial to anticipate the geometry associated with the interaction of gold nanorods and magnetic nanoparticles. TEM images also allowed to confirm the formation of the magneto-plasmonic nanostructures. From Figure 3e, it is possible to observe the coupling of gold nanorods with magnetic nanoparticles.

**Figure 4:**
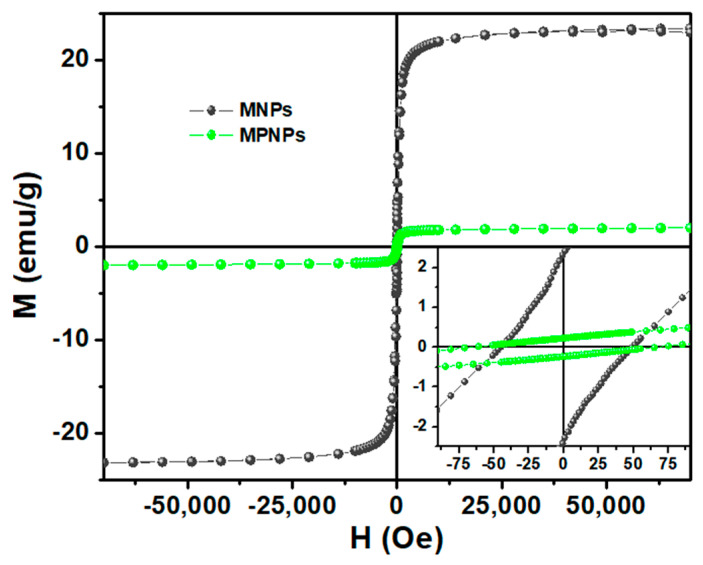
Magnetization hysteresis loops of Mg_0.75_Ca_0.25_Fe_2_O_4_ magnetic nanocubes (MNPs) before (grey) and after (green) coupling with gold nanorods (MPNPs), measured at T = 300 K. The inset provides a magnified view of the M-H loops in the low field region.

The magnetic behavior of both MNPs and MPNPs was evaluated by SQUID, aiming to study the effect of the plasmonic component in the magnetic properties. Figure 4 shows the dependence of the magnetic moment (M) on the applied magnetic field (H), at room temperature. The coercive field (H_c_), saturation magnetization (M_s_), remnant magnetization (M_r_) and the ratio between M_r_/M_s_ (squareness value) were obtained and are summarized in Table 1.

The obtained results clearly emphasize that the M–H curve of cubic-shaped magnetic NPs was significantly influenced after coupling with the gold nanorods due to their diamagnetic contribution. Although both hysteresis cycles display some attributes typical of particles with a superparamagnetic behavior, such as an almost closed loop and low remnant magnetization (2.4 and 0.23 emu/g for MNPs and MPNPs, respectively), the M_s_ was highly compromised after the coupling with the plasmonic NPs. This parameter decreased from 23.5 to 2.2 emu/g, representing a reduction of more than tenfold. Even so, the squareness value of, approximately, 0.1 for both NPs points to a superparamagnetic nature at room temperature, indicating that 90% of the magnetization is lost upon the removal of the applied magnetic field [49].

**Figure 5:**
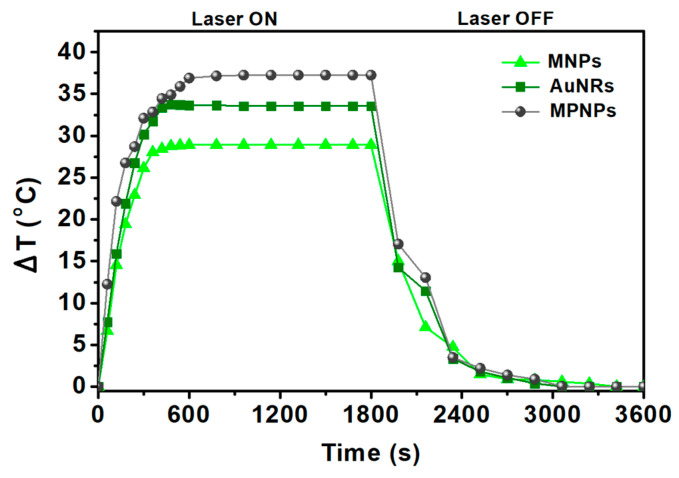
Heating profile of aqueous dispersions (1 mg/mL) of Mg_0.75_Ca_0.25_Fe_2_O_4_ magnetic nanocubes (MNPs), gold nanorods (AuNRs) and magnetic/plasmonic nanoparticles (MPNPs) under exposure to a laser light source with 808 nm wavelength and 1 W/cm^2^ power density. ΔT is the increment from room temperature (20 °C).

The photothermic potential of the prepared nanoparticles was assessed using NIR irradiation. In these measurements, each type of nanoparticle was evaluated separately. Thus, aqueous dispersions of neat MNPs, neat AuNRs and coupled MPNPs, at the same NP concentration (1 mg of NPs per mL of water), were prepared. For comparison, pure water was also tested to evaluate the heating of the medium in the absence of nanoparticles. The heating and cooling curves are shown in Figure 5.

SAR values were obtained using the initial slope method. The linear region of the curve covers the time during which the heating process occurs effectively, without any loss/exchange of heat with the environment, making it possible to extract the actual temperature increase caused by laser irradiation. The obtained results are summarized in Table 2.

As expected, the gold nanorods showed better heating capacity compared to the magnetic nanoparticles, with corresponding SAR values of 523.8 W/g and 467.9 W/g, for AuNRs and MNPs, respectively. Regarding magnetic/plasmonic NPs, these exhibited a higher SAR when compared to individual components. This favorable behaviour shows the synergistic effect between the plasmonic and magnetic components in the nanosystem, potentiating the photothermal capability. The MPNP sample would still contain 68% of MNPs and some AuNRs arrested in the centrifugation pellet. However, the conclusion regarding the synergistic effect is the same and purification of MPNP samples would have resulted in an even higher SAR. This synergistic effect has been reported previously for gold nanorods and magnetite nanoparticles in a silica matrix [26,50], although a clear reasoning for such behavior was not presented and is still under investigation. One possibility is an increased intensity of the plasmon resonance in the NIR region (as can be observed in Figure 2e), if one takes into account the 32% coupling efficiency. This increase has been reported in theoretical calculations on related systems with a layer-by-layer geometry [51]. As MgFe_2_O_4_ is a known n-type semiconductor [52], another possibility is an efficient charge transfer from electrons in MgFe_2_O_4_ to gold, due to the close proximity of the two nanoparticles. Moreover, the maximum temperature variation is adequate for therapeutic action, since the temperature of photothermal hyperthermia (42–45 °C) is easily reached at physiological temperature.

### 3.2. Magneto-Plasmonic Elastic Liposomes’ Characterization

#### 3.2.1. Elastic Properties Investigation

The prepared nanoparticles were encapsulated in elastic liposomes to ensure the absorption of the nanoparticles into the skin. For topical applications, the thermosensitive phospholipid DPPC is preferred as the main component, in consequence of its low T_m_ (41 °C) [10]. To destabilize the vesicles and increase their deformability, SP60, SP65, SP80, SP85 and TW80 were used as EAs. Thus, the impact of the lipid composition on the degree of elasticity of the ELs was assessed using fluorescence anisotropy studies. For this, the membrane probe DPH was included into the lipid bilayer. This probe is widely used to monitor the membrane fluidity of biological systems, being an indirect reporter of their microviscosity [40]. Several formulations with different compositions were investigated regarding their micro-viscosity at 25 °C (room temperature) and 37 °C (body average temperature [53]). These temperature points were selected, as they fall below and above the average skin temperature range (between 33.5 °C and 36.9 °C) [54]. Considering that η is indirectly indicative of the degree of elasticity, it was assumed that the lower its value, the higher is the liposomes’ fluidity and, consequently, their elasticity. Table 3 summarizes the results of the anisotropy experiments, using equation (4) for micro-viscosity estimation (considering that the variation of DPH fluorescence lifetime between 25 °C and 37 °C is very low [55] and could be neglected).

These data clearly indicate a correlation between the liposomes’ microviscosity and the type of surfactant. Although to dissimilar degrees and excluding DPPC:TW80 (80:20) liposomes, it can be inferred that all the developed formulations exhibit elastic properties at 25 °C, as their fluidity increased in comparison to the system composed only by DPPC. Despite the variations observed in the three ratios, liposomes containing Tween-80 as edge activator are too viscous, potentially hindering their ability to cross the barriers of the skin. On the other hand, formulations composed by SP80 and SP85 have shown more potential as ELs, exhibiting the lowest η values at both temperatures. Considering the aim of this work, liposomes of DPPC:SP80 (85:15) were selected to encapsulate the MPNPs. This formulation stands out for its reduced microviscosity at 37 °C (1.3 ± 0.3 P), being that with the highest degree of elasticity. In this way, the loaded vesicles will be able to “squeeze” and pass through the skin pores by modulating its structure. In fact, Hussain et al. [9] proved that liposomes containing SP80 have an adequate vesicular size and high elasticity. Moreover, this formulation showed the highest drug permeation flux (89.74 ± 8.5 mg/cm^2^/h) and proved to be able to deliver the drug 5-fluorouracil into the SC for enhanced skin delivery with minimal hemolysis. These results, together with those obtained here, point to the development of novel MPELs of DPPC:SP80 (85:15) loaded with magneto-plasmonic NPs.

#### 3.2.2. Study of the Phase Transition Temperature of MPELs

**Figure 6:**
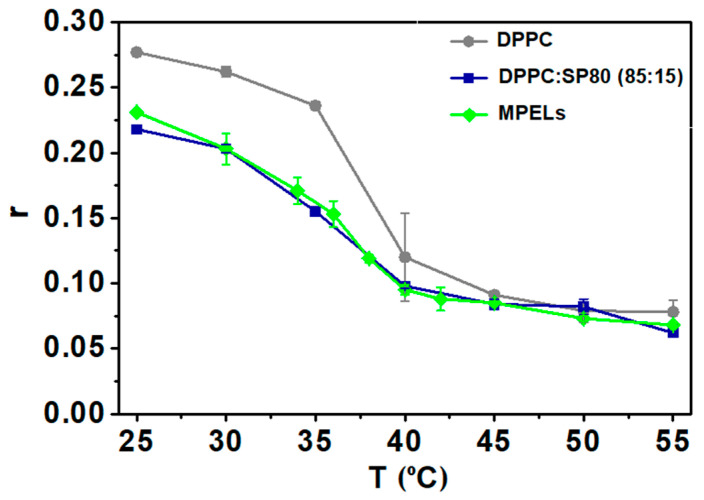
Temperature dependence of fluorescence anisotropy (r) of DPH incorporated into liposomes of DPPC:SP80 (85:15) and magneto-plasmonic elastic liposomes (MPELs). DPPC liposomes are shown for comparison.

As mentioned in Section 1, it is important to use edge activators as an ingredient to prepare ELs, considering that these play a crucial role in decreasing the phase transition temperature of typical liposomes and conferring them elastic properties. In this context, anisotropy studies were conducted in order to identify the transition temperature of the nanosystems and understand the influence of EAs on this parameter. For this assay, DPH fluorescence anisotropy was used to predict the properties of lipid bilayers at different temperatures (Figure 6).

Liposomes composed only of DPPC exhibit a T_m_ around 41 °C, as previously reported [40,55]. Similar results were obtained for fluorescence anisotropy of DPH in neat DPPC liposomes, as reported by Pereira et al. [56]. It is particularly highlighted that the inclusion of Span-80 into lipid vesicles is actually reflected in a notable decrease of the probe’s fluorescence anisotropy, which indicates an increase in membrane fluidity. This observation validates the effectiveness of SP80 as edge activator in reducing the melting temperature of liposomes and, consequently, in promoting their elastic nature. Regarding MPELs, it is concluded that magneto-plasmonic NPs did not induce any significant changes in the system’s phase transition temperature, of around 35 °C (inflexion point of the curve). As the liposomes’ T_m_ should be localized in the range 25 °C to 35 °C, an improved permeation flux is assured when in direct contact with the skin. At this temperature, MPELs will adopt a more fluidic structure (liquid crystal state) capable of crossing the SC through the intercellular route, ensuring the “same time at same place” therapeutic strategy.

The recognition of the system’s phase transition temperature is also of major interest to understand its role as mediator of cellular damage via phototherapy. For this type of therapeutic modality, temperatures in the range of 42 °C to 45 °C are generally effective in starting to accelerate biochemical reactions, leading to protein denaturation and production of reactive oxygen species (ROS) [57]. The T_m_ of the developed magneto-plasmonic liposomes suggests that these cytotoxic episodes would be triggered only upon penetration into the skin layers. It is expected that, when in contact with the skin at 35 °C, the fluidity of MPELs increases, allowing them to cross this barrier. By focusing a NIR laser light in the target site, a localized temperature rise would be promoted and, upon reaching that thermal interval, apoptotic events should be exploited [57]. Additionally, a thermosensitive nano-system with this T_m_ would be capable of retaining drugs at physiological conditions and selectively release their content at the target location only after penetration and reaching hyperthermic temperatures.

#### 3.2.3. MPELs’ Structural Characterization and Stability

**Figure 7:**
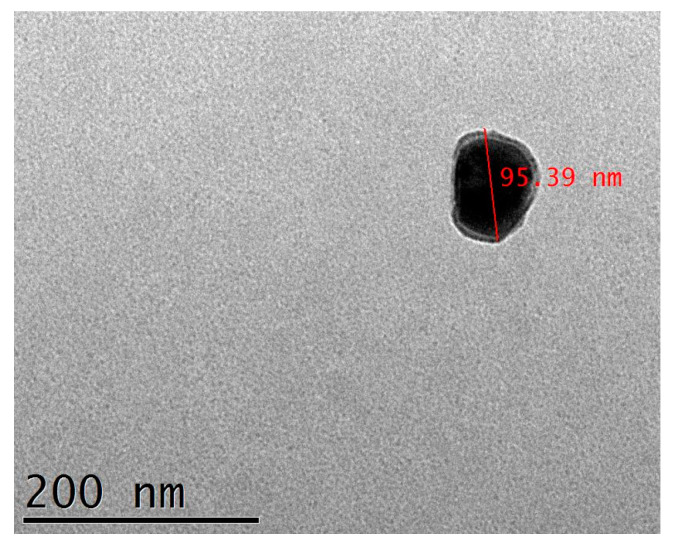
TEM image of DPPC:SP80 (85:15) magneto-plasmonic elastic liposomes (scale bar: 200 nm).

Here, magneto-plasmonic elastic liposomes were prepared for the first time using the conventional ethanolic injection method. This technique was chosen for its easy synthesis, while producing homogeneous, monodisperse and stable solutions of small uni-lamellar vesicles without the need of additional steps as extrusion, that can lead to the liposomes’ degradation and oxidation [58]. The TEM image of MPELs (Figure 7) shows structures with sizes of around 95 nm, with a dark core (the magneto-plasmonic core after the application of vacuum) surrounded by a thin layer (the lipid bilayer). The polydispersity index (PDI) below 0.3 (0.25 ± 0.015), measured by DLS, indicates generally monodisperse structures. For lipid-based nanocarriers, suspensions with standard PDI values below 0.3 are considered acceptable for biomedical applications [59]. MPELs size between 85 and 95 nm are in the defined range for biomedical applications (~50–200 nm) [60], making them promising candidates for the main objective of this work. Moreover, the liposomes’ zeta potential (ζ), before and after the magneto-plasmonic NPs incorporation (−1.18 ± 0.157 eV for ELs and +19.56 ± 1.039 eV for MPELs), reveal that being a non-ionic surfactant SP80 did not cause changes in the near-neutral charge of the liposomes conferred by the zwitterionic lipid DPPC. However, the stability of the nanosystem was modified by adding the hyperthermia agent. The positive surface charge obtained in magneto-plasmonic elastic liposomes is a result of the capping agent used in the preparation of AuNRs. CTAB is a cationic surfactant that acts in the stabilization of nanorods during their synthesis forming a bilayer structure around the particles [61]. In addition, the obtained positive charge suggests that the magneto-plasmonic NPs must be located close to the surface of the liposomes.

**Figure 8:**
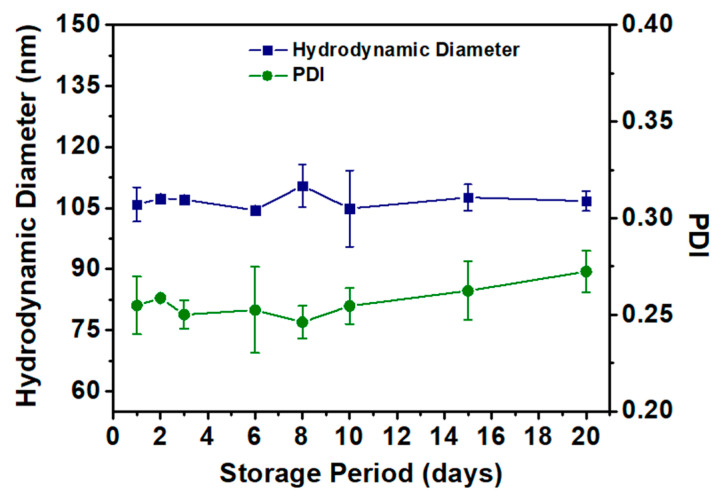
Stability of MPELs aqueous solution stored at 4 °C, represented as the variation of its hydrodynamic diameter and PDI over a 20-day period.

A stability study was performed to determine whether these magneto-plasmonic elastic liposomes satisfy the requirements for commercial and clinical outcome. For that purpose, mean size and PDI variations were monitored over a 20-day storage period at 4 °C (Figure 8); considerable changes in these domains reveal signs of either aggregation or membrane rupture [62]. During this time, the MPELs size presented minimal shifts from the day 1 value (D_0_ = 105.9 ± 4 nm; D_20_ = 106.7 ± 3 nm), suggesting that these should remain stable in long-term storage. In addition, no significant PDI deviations were found. Still, the MPELs’ suspension shows an increasing pattern in that index, starting from the 8th day onward. With this background, the developed nanostructure is expected to, when in contact with the skin, easily adjust its shape and be rapidly absorbed, permeating the SC up to, at least, 20 days after synthesis.

#### 3.2.4. MPELs as Photothermal Hyperthermia Agents

**Figure 9:**
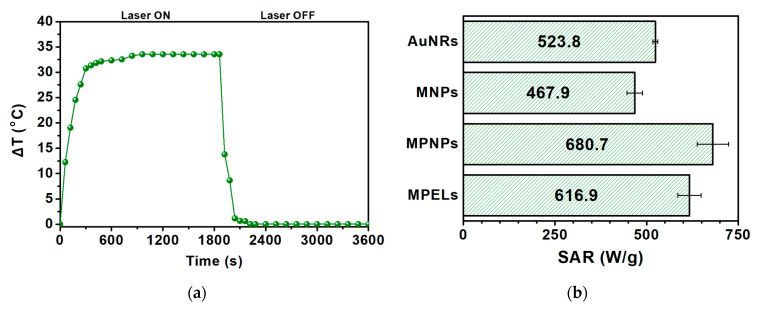
(**a**) Heating profile of an aqueous solution of magneto-plasmonic elastic liposomes under exposure to a laser light source with 808 nm wavelength and 1 W/cm^2^ power density. ΔT is the increment from room temperature (20 °C). (**b**) Histogram of specific absorption rate, SAR (W/g), of gold nanorods (AuNRs), Mg_0.75_Ca_0.25_Fe_2_O_4_ magnetic nanocubes (MNPs), magnetic/plasmonic nanoparticles (MPNPs) and magneto-plasmonic elastic liposomes (MPELs).

The photothermal potential of the novel MPELs here developed was assessed under laser exposure at 808 nm. This specific wavelength is frequently employed in such experiments, as it falls within the first near-infrared (NIR-I) biological window (700–900 nm), i.e., spectral wavelengths capable of penetrating biological tissues with minimum absorbance and scattering [16]. Under these conditions, the ability for energy dissipation of magnetic/plasmonic NPs enclosed in elastic liposomes of DPPC:SP80 (85:15) was assessed (for the same concentration of MPNPs used before). The equivalent SAR and temperature variation (above room temperature), 616.9 ± 32 W/g and 33.5 °C, respectively (Figure 9), lead to a conclusion that the final nanostructure exhibits appealing features for performing efficient photothermal hyperthermia to treat topical pathologies.

## 4. Conclusions

This research aimed to design and prepare an innovative nanoplatform with ultra-deformable properties for applications in skin cancer diseases by photothermal hyperthermia. For that purpose, gold nanorods and cubic-shaped magnetic nanoparticles of magnesium ferrite with 25% replacement by calcium ions were synthetized and characterized through XRD, UV-Vis–NIR spectroscopy, TEM and SQUID. TEM images validated the synthesis, coupling and encapsulation of magneto-plasmonic nanoparticles into DPPC:SP80 (85:15) thermoelastic liposomes, with suitable properties for biomedical applications. Photothermal hyperthermia measurements under a NIR laser light demonstrated an encouraging prospect of MPELs in addressing topical disease conditions (SAR = 617 W/g). Therefore, this work provides valuable insights into the potential of the developed nanosystems for future potential application in skin cancer.

To the best of our knowledge, this is the first time that magneto-plasmonic elastic liposomes containing Ca/Mg ferrites coupled to gold nanorods were developed and characterized, and their photothermal capability evaluated.

## Figures and Tables

**Table 1:** Coercive field, H_c_ (Oe), saturation magnetization, M_s_ (emu/g), remnant magnetization, M_r_ (emu/g), and M_r_/M_s_ ratio for magnetic (MNPs) and magneto-plasmonic nanoparticles (MPNPs) at room temperature.

Samples	H_c_ (Oe)	M_s_ (emu/g)	M_r_ (emu/g)	M_r_/M_s_
MNPs	49.0	23.5	2.4	0.102
MPNPs	67.2	2.20	0.23	0.105

**Table 2:** Specific absorption rate, SAR (W/g), for gold nanorods (AuNRs), Mg_0.75_Ca_0.25_Fe_2_O_4_ magnetic nanocubes (MNPs) and magneto-plasmonic nanoparticles (MPNPs), under a NIR laser light (808 nm wavelength and 1 W/cm^2^ power density).

Sample	SAR (W/g)	ΔT (°C)
AuNRs	523.8 ± 6.7	33.6
MNPs	467.9 ± 21	28.9
MPNPs	680.7 ± 43	37.2

**Table 3:** Fluorescence anisotropy, r, of DPH and micro-viscosity, η (P), of several lipid formulations at 25 °C and 37 °C.

		Temperature			
		25 °C		37 °C	
Formulation	Ratio	r	η (P)	r	η (P)
DPPC	---	0.277 ± 0.003	7.9 ± 0.3	0.236 ± 0.003	4.5 ± 0.2
DPPC:SP60	70:30	0.261 ± 0.015	6.4 ± 1.2	0.209 ± 0.020	3.3 ± 0.8
	80:20	0.249 ± 0.014	5.5 ± 1	0.242 ± 0.005	4.9 ± 0.3
	85:15	0.252 ± 0.005	5.5 ± 0.3	0.232 ± 0.014	4.4 ± 0.7
DPPC:SP65	70:30	0.222 ± 0.004	3.8 ± 0.2	0.197 ± 0.004	2.9 ± 0.1
	80:20	0.243 ± 0.003	4.9 ± 0.2	0.223 ± 0.019	3.9 ± 0.8
	85:15	0.230 ± 0.004	4.2 ± 0.2	0.226 ± 0.008	4.0 ± 0.5
DPPC:SP80	70:30	0.225 ± 0.015	4.0 ± 0.6	0.135 ± 0.002	1.4 ± 0.03
	80:20	0.248 ± 0.007	5.3 ± 0.5	0.144 ± 0.002	1.6 ± 0.04
	85:15 *	0.218 ± 0.003	3.63 ± 0.01	0.125 ± 0.018	1.3 ± 0.3
DPPC:SP85	70:30	0.207 ± 0.005	3.2 ± 0.2	0.112 ± 0.004	1.6 ± 0.05
	80:20	0.230 ± 0.010	4.2 ± 0.5	0.107 ± 0.002	1.3 ± 0.03
	85:15	0.231 ± 0.018	4.4 ± 0.9	0.135 ± 0.024	1.5 ± 0.4
DPPC:TW80	70:30	0.274 ± 0.006	7.5 ± 0.7	0.251 ± 0.002	5.4 ± 0.1
	80:20	0.282 ± 0.003	8.5 ± 0.4	0.236 ± 0.011	4.5 ± 0.5
	85:15	0.269 ± 0.015	7.2 ± 1	0.224 ± 0.008	3.9 ± 0.4

* Selected formulation.

## Data Availability

Data available upon request.
